# Direct comparison of diagnostic and clinical values between Tc-99 m DPD and Tc-99 m PYP scintigraphy in patients with cardiac amyloidosis

**DOI:** 10.1186/s12880-023-01054-x

**Published:** 2023-07-17

**Authors:** Yong-Jin Park, Joohee Lee, Darae Kim, Jin-Oh Choi, Seok Jin Kim, Kihyun Kim, Joon Young Choi

**Affiliations:** 1 Department of Nuclear Medicine, Samsung Medical Center, Sungkyunkwan University School of Medicine, 81, Irwon-ro, Gangnam-gu, Seoul, 06351 Republic of Korea; 2 Department of Nuclear Medicine, Ajou University Medical Center, Ajou University School of Medicine, Suwon, 16499 Republic of Korea; 3 Department of Nuclear Medicine, CHA Ilsan Medical Center, CHA University, Goyang, 10414 Republic of Korea; 4 Division of Cardiology, Department of Medicine, Heart Vascular Stroke Institute, Samsung Medical Center, Sungkyunkwan University School of Medicine, Seoul, 06351 Republic of Korea; 5 Division of Hematology-Oncology, Department of Medicine, Samsung Medical Center, Sungkyunkwan University School of Medicine, Seoul, 06351 Republic of Korea

**Keywords:** Cardiac amyloidosis, Tc-99m DPD, Tc-99m PYP, Perugini system, Dorbala system

## Abstract

**Background:**

Technetium-99 m 3,3-diphosphono-1,2-propanodicarboxylic acid (DPD) and technetium-99 m sodium pyrophosphate (PYP) are the two most commonly used radiotracers for cardiac amyloidosis (CA), but no studies have directly compared them. Therefore, in this study, we directly compared the diagnostic and clinical utility of DPD and PYP scintigraphy in patients with CA.

**Methods:**

Ten patients with CA were enrolled. Eight clinical variables and 12 scintigraphic parameters were used. Clinical variables were age, sex, estimated glomerular filtration rate (eGFR), N-terminal pro brain natriuretic peptide (NT-proBNP), and the results of electromyography (EMG), a sensory test, electrocardiogram, and echocardiography (EchoCG). Four heart retention ratios (heart/whole-body profile, heart/pelvis, heart/skull, and heart/contralateral lung) were calculated from the DPD and PYP scans and two visual scoring systems (Perugini and Dorbala systems) were used. Comparative analyses were performed between radiotracers and between visual scoring systems using clinical variables and scintigraphic parameters.

**Results:**

Twenty DPD parameters and nine PYP parameters had significant associations with age, eGFR, NT-proBNP, EchoCG, and EMG. DPD parameters had more frequent significant associations with clinical variables than PYP parameters. Compared to visual scores in the DPD scan, the proportion of patients with higher visual scores in the PYP scan was relatively greater than those with lower visual scores, and there were more patients with a visual score of 2 or higher in PYP scans than DPD scans.

**Conclusions:**

DPD scintigraphy may reflect the disease severity of CA better than PYP scintigraphy, whereas PYP scintigraphy may be a more sensitive imaging modality for identifying CA involvement.

**Supplementary Information:**

The online version contains supplementary material available at 10.1186/s12880-023-01054-x.

## Background

Cardiac amyloidosis (CA) is one of the most common restrictive infiltrative cardiomyopathies (CMs). It has a poor prognosis, with the clinical outcome dependent on the type of amyloid fibril deposition and the extent of organ and tissue involvement [1]. Various types of amyloidosis can affect the heart, but the two most common types are dominant: amyloid transthyretin (ATTR) amyloidosis and amyloid immunoglobulin light-chain (AL) amyloidosis [2, 3]. Timely diagnosis of CA is crucial given the effectiveness of newly available treatments [2]. Known non-invasive diagnostic methods for CA include electrocardiogram (ECG), echocardiography (EchoCG), cardiac biomarkers, cardiovascular magnetic resonance (CMR), and radiotracers for nuclear medicine imaging [4]. Technetium-99 m phosphate derivatives including technetium-99 m 3,3-diphosphono-1,2-propanodicarboxylic acid (DPD), technetium-99 m sodium pyrophosphate (PYP), and technetium-99 m hydroxymethylene diphosphonate are appropriate for evaluating ATTR CA [5].

Among radiotracers for cardiac scintigraphy, DPD and PYP are two most commonly used radiotracers for ATTR CA [6]. In a previous study of patients with CA identified using DPD scintigraphy, all patients with ATTR CA showed moderate to severe DPD uptake, whereas all patients with AL CA showed no or mild DPD uptake [7]. In a previous study in patients with genetically verified hereditary ATTR CM using DPD single-photon emission computed tomography (SPECT)/computed tomography (CT), DPD-based amyloid burden showed a robust correlation with logarithmic N-terminal pro brain natriuretic peptide (NT-proBNP) and troponin T levels [8]. Takasone et al. reported that all patients with wild-type ATTR CA showed positive PYP uptake, whereas all patients with AL CA showed negative PYP uptake. In a previous study using PYP SPECT/CT, the maximum standardized uptake value (SUV) of ATTR CM was significantly higher than that of AL CM [9]. To date, various studies on CA using DPD or PYP imaging have been published, but no study has directly compared DPD and PYP scans.

Several scintigraphic parameters have been proposed for cardiac scintigraphy with bone-seeking radiotracers. In semi-quantitative analyses, Gallini et al. used six indices to identify CA patients [10]: heart/whole-body (H/WB) rectangular region of interest (ROI) ratio, heart/whole-body profile (H/WBp) ROI ratio, heart/pelvis (H/P) ratio, H/P ratio with tissue background correction, heart/skull (H/S) ratio, and heart/contralateral lung (H/CL) ratio. These authors suggested that H/WB ratios may be effective and strong semi-quantitative indices for evaluating CA. Based on qualitative analyses, Kessler et al. reported that the Perugini visual scoring system of DPD scintigraphy and SPECT/CT was significantly correlated with cardiac maximum SUV in patients with suspected CA [11]. In a previous PYP SPECT/CT study in patients with suspected CA, the Dorbala visual scoring system had a strong positive correlation with extracellular volume on CMR [12]. Bae et al. suggested that quantitative indices had stronger correlations with the Dorbala system than with the Perugini system in patients with suspicion of CA based on DPD scans [13].

Our aim in this study was to directly compare DPD and PYP scintigraphy in patients with CA to evaluate their diagnostic and clinical utilities. For comparative analyses, the scintigraphic parameters of four heart retention ratios (H/WBp, H/P, H/S, and H/CL) and two visual scoring systems (Perugini and Dorbala) were used. In addition, the clinical value of each visual scoring system was investigated.

## Materials and methods

### Study population

We retrospectively reviewed the medical records of patients with CA diagnosed at Samsung Medical Center between August 2017 and July 2020. All study patients underwent both DPD and PYP scintigraphy, and the period between DPD and PYP scintigraphy was less than six months. Experienced cardiologists (D.K. and J.-O.C.) diagnosed patients with CA based on histopathological, genetic, cardiac-related, and hematologic findings. Endomyocardial or extracardiac biopsies were taken to identify amyloidosis, and immunohistochemistry staining was performed using these tissue specimens. Transthyretin (TTR) gene analysis was performed to confirm TTR gene mutations using deoxyribonucleic acid extracted from peripheral blood. CMR, EchoCG, ECG, DPD and PYP scintigraphy, cardiac biomarker levels, and cardiac complications and comorbidities were evaluated to assess cardiac-related findings. Hematologic findings were evaluated based on serum free-light chain quantification and serum and urine protein electrophoresis with immunofixation. Cardiologists diagnosed patients with CA using invasive and non-invasive diagnostic criteria for CA based on the histopathological, genetic, cardiac-related, and hematologic findings described above [14]. Finally, 10 patients diagnosed CA were enrolled; nine were diagnosed with TTR CA and one with AL CA.

The Institutional Review Board (IRB) of Samsung Medical Center approved this retrospective study (IRB number: 2022-08-111). Informed consent was waived by IRB of Samsung Medical Center due to the retrospective nature of the study. This study was conducted according to the Declaration of Helsinki 2013 and its later amendments or similar ethical standards.

### DPD and PYP scintigraphy

We used the same acquisition protocol for DPD and PYP scintigraphy. Three hours after intravenous injection of DPD or PYP (740 MBq), anterior and posterior whole-body (WB) scans were performed using a dual-headed gamma camera (e.cam, Siemens Medical Systems) equipped with a low-energy, high-resolution collimator. The matrix size of images was set to 256 × 1024. Scan speed and scan length were 20 cm/min and 195 cm, respectively.

### Image analysis

An experienced nuclear medicine physician (J.L.) interpreted and analyzed WB planar images using Xeleris Functional Imaging Workstation version 4.0 (GE Healthcare, Milwaukee, WI, USA). Two visual scoring systems, Perugini and Dorbala [12, 15, 16], were used to visually confirm myocardial radiotracer uptake. In the Perugini system, a score of 0 was defined as no cardiac retention uptake and normal bone uptake, while a score of 1 indicated mild cardiac retention uptake and inferior bone uptake. A score of 2 was defined as moderate cardiac retention uptake accompanied by attenuated bone uptake, and a score of 3 indicated strong cardiac retention uptake with absent/mild bone uptake [16]. In the Dorbala system, similar to the Perugini system, a score of 0 was defined as no cardiac retention uptake and normal bone uptake, a score of 1 indicated cardiac retention uptake less than rib uptake, a score of 2 was defined as cardiac retention uptake equal to rib uptake, and a score of 3 indicated cardiac retention uptake greater than rib uptake with absent/mild rib uptake [12, 15].

**Fig. 1:**
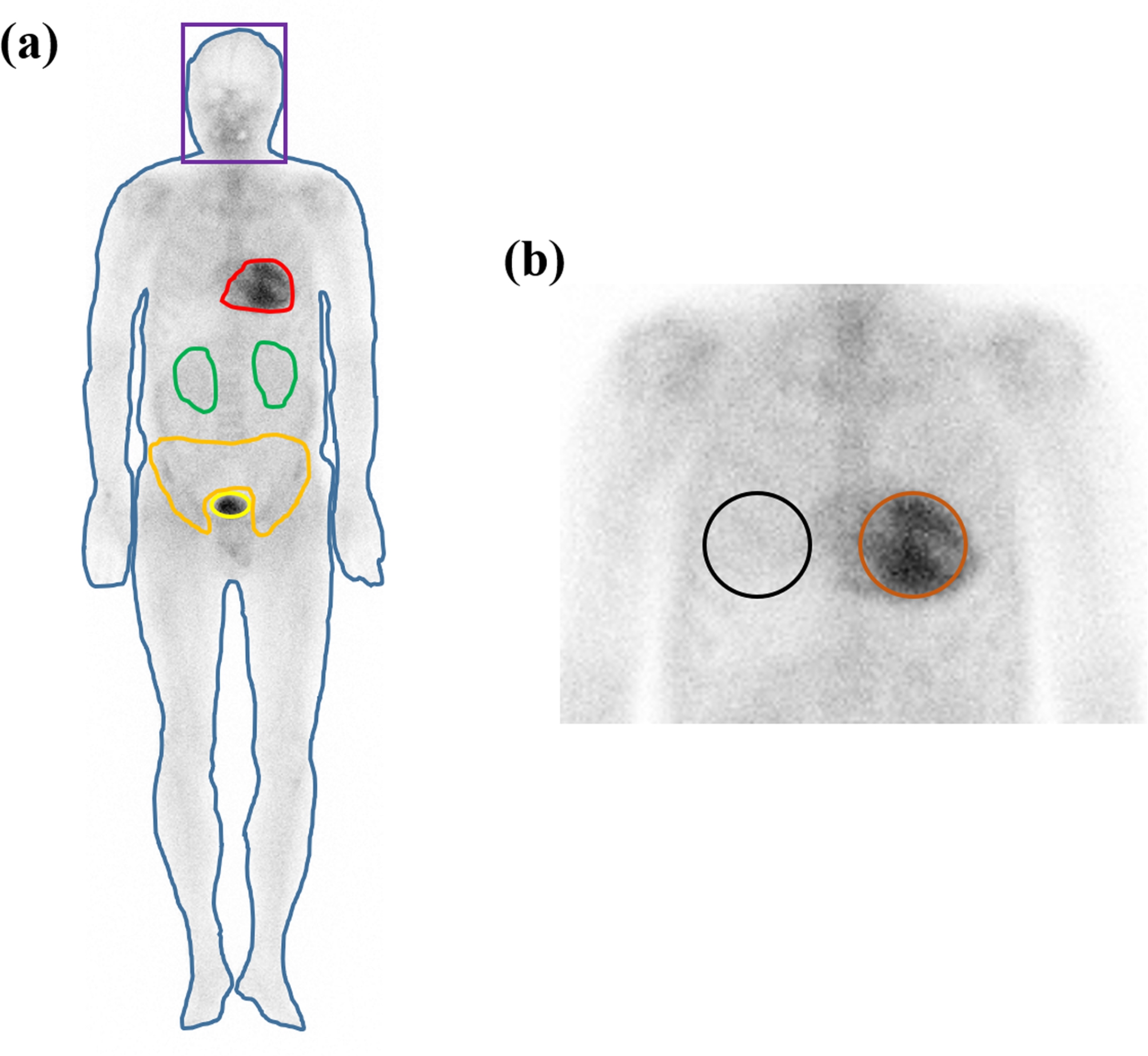
ROI drawing methods for heart retention ratios. **(a)** WB profile, heart, kidney, bladder, and pelvis ROIs were drawn manually. In addition, the skull ROI was drawn using a rectangular ROI. H/WBp, H/P, and H/S ratios were calculated using these ROIs. **(b)** To calculate the H/CL ratio, heart and contralateral lung ROIs were drawn using simplified circular ROIs Abbreviations: ROI, region of interest; WB, whole-body; H/WBp, heart/whole-body profile ratio; H/P, heart/pelvis ratio; H/S, heart/skull ratio; H/CL, heart/contralateral lung ratio

Four semi-quantitative heart retention ratios were calculated on WB planar images using ROIs based on a previous study (Fig. 1) [10]. The four heart retention ratios were as follows: H/WBp, H/P, H/S, and H/CL. For the H/WBp ratio, a WB ROI was drawn along the WB profile on the anterior projection. In addition, heart, kidney, and bladder ROIs were drawn on the anterior projection, and radiotracer uptake was quantified as the total count in each ROI. H/WBp was calculated by the dividing heart count by the WB count minus the counts of both kidneys and the bladder. For the H/P ratio, heart and pelvis ROIs were drawn on the anterior projection, and the pelvis ROI was defined as the anatomical ROI around the sacrum and pelvis profile. The H/P ratio was calculated by dividing the heart count by the pelvis count. For the H/S ratio, heart and skull ROIs were drawn on the anterior and posterior projections, and skull ROIs were drawn using rectangular ROIs around the skull. Radiotracer uptake was quantified as the mean count for each ROI on anterior and posterior projections, and the H/S ratio was calculated by dividing the mean heart count by the mean skull count. For the H/CL ratio, heart and specular contralateral lung (CL) ROIs were drawn using simplified circular ROIs on the anterior projection. The H/CL ratio was calculated by dividing the heart count by the CL count.

### Clinical variables and scintigraphic parameters

In this study, eight clinical variables and 12 scintigraphic parameters were used. Clinical variables were age, sex, estimated glomerular filtration rate (eGFR), NT-proBNP, and the results of electromyography (EMG), a quantitative sensory test, ECG, and EchoCG. Clinical variables were assessed within 3 months of initial diagnosis of CA. Among these, the results of EMG, sensory test, ECG, and EchoCG were converted into dichotomous variables according to abnormal findings. In EMG, a sympathetic postganglionic sudomotor dysfunction was considered an abnormal finding. In ECG, atrioventricular block, atrial fibrillation, ventricular arrhythmia, and sinus bradycardia were considered abnormal findings. In the quantitative sensory test, abnormal findings were those that exceeded the cooling and vibration thresholds. EchoCG results were classified into two categories according to diastolic dysfunction; a diastolic dysfunction grade of 1 or higher was considered an abnormal finding. Twelve scintigraphic parameters comprised eight retention ratios and four visual scores in DPD and PYP scans. The eight heart retention ratios were DPD H/WBp, DPD H/P, DPD H/S, DPD H/CL, PYP H/WBp, PYP H/P, PYP H/S, and PYP H/CL. The four visual scores were DPD Perugini, DPD Dorbala, PYP Perugini, and PYP Dorbala.

### Statistical analysis

Comparative analyses were performed between radiotracers and between visual scoring systems using clinical variables and scintigraphic parameters. The Shapiro-Wilk test was used to assess the normality of continuous variables. Mann-Whitney U test was used to compare differences between dichotomous variables. Fisher’s exact test was used to assess nonrandom associations between two discrete dichotomous variables. Spearman’s correlation was used to measure the strength of linear relationship between two continuous variables. Statistical analyses were performed using MedCalc statistical software (version 20.110, Ostend, Belgium). All statistical tests were two-tailed, and a P value < 0.05 was considered statistically significant.

## Results

### Patient characteristics

In this study, 10 patients diagnosed with CA were enrolled (Table 1). Nine (90%) were diagnosed with ATTR CA, while one patient (10%) was diagnosed with AL CA. Five patients, three patients, and one patient had heart, soft tissue, and nerve biopsies, respectively, and these nine patients were confirmed to have amyloidosis. The one patient who did not undergo biopsy was confirmed to have a TTR gene mutation. Of the nine patients diagnosed with ATTR CA, all patients had positive immunohistochemical staining for Congo red and TTR. In addition, eight out of nine patients diagnosed with ATTR CA underwent TTR gene analysis, and all were found to have mutations in TTR. Identified variants were p.Asp58Ala, p.Met33dup, p.Asp119Asn, p.Glu109Lys, and p.Thr79Lys, with p.Asp58Ala the most common variant (n = 4). The patient diagnosed with AL CA had negative staining for TTR and positive staining for Congo red, amyloid P, lambda, and kappa in immunohistochemistry staining. Additionally, this patient was confirmed to have no TTR gene mutations but to have a monoclonal gammopathy and a Perugini score of 2 and Dorbala score of 3 in PYP scintigraphy. Of the 10 patients enrolled, six (60%) were male. Median age, eGFR, and NT-proBNP were 56 years (range of 41–68 years), 99 mL/min/1.74 m^2^ (range of 67–115 mL/min/1.74 m^2^), and 1,212 pg/mL (range of 31 − 6,030 pg/mL), respectively. Among the study patients, more than half of the patients had abnormal EMG (80%) and sensory test (90%) findings, and half of the patients had abnormal ECG findings (50%). Eight (80%) patients had diastolic grade 1 or higher on EchoCG.

**Table 1:** Patient characteristics

		Median (range) or number of patients (%)
Age (years)		56 (41–68)
Sex (male)		6 (60%)
eGFR (mL/min/1.74 m^2^)		99 (67–115)
NT-proBNP (pg/mL)		1,212 (31 − 6,030)
EMG (abnormal)		8 (80%)
Sensory test (abnormal)		9 (90%)
ECG (abnormal)		5 (50%)
EchoCG	Diastolic dysfunction grade (0/1/2/3)	2/3/2/3
	LVEF (%)	55 (31–69)

Abbreviations: eGFR, estimated glomerular filtration rate; NT-proBNP, N-terminal pro brain natriuretic peptide; EMG, electromyography; ECG, electrocardiogram; EchoCG, echocardiography; LVEF, left ventricular ejection fraction

### Comparisons of scintigraphic parameters between DPD and PYP scans

**Fig. 2:**
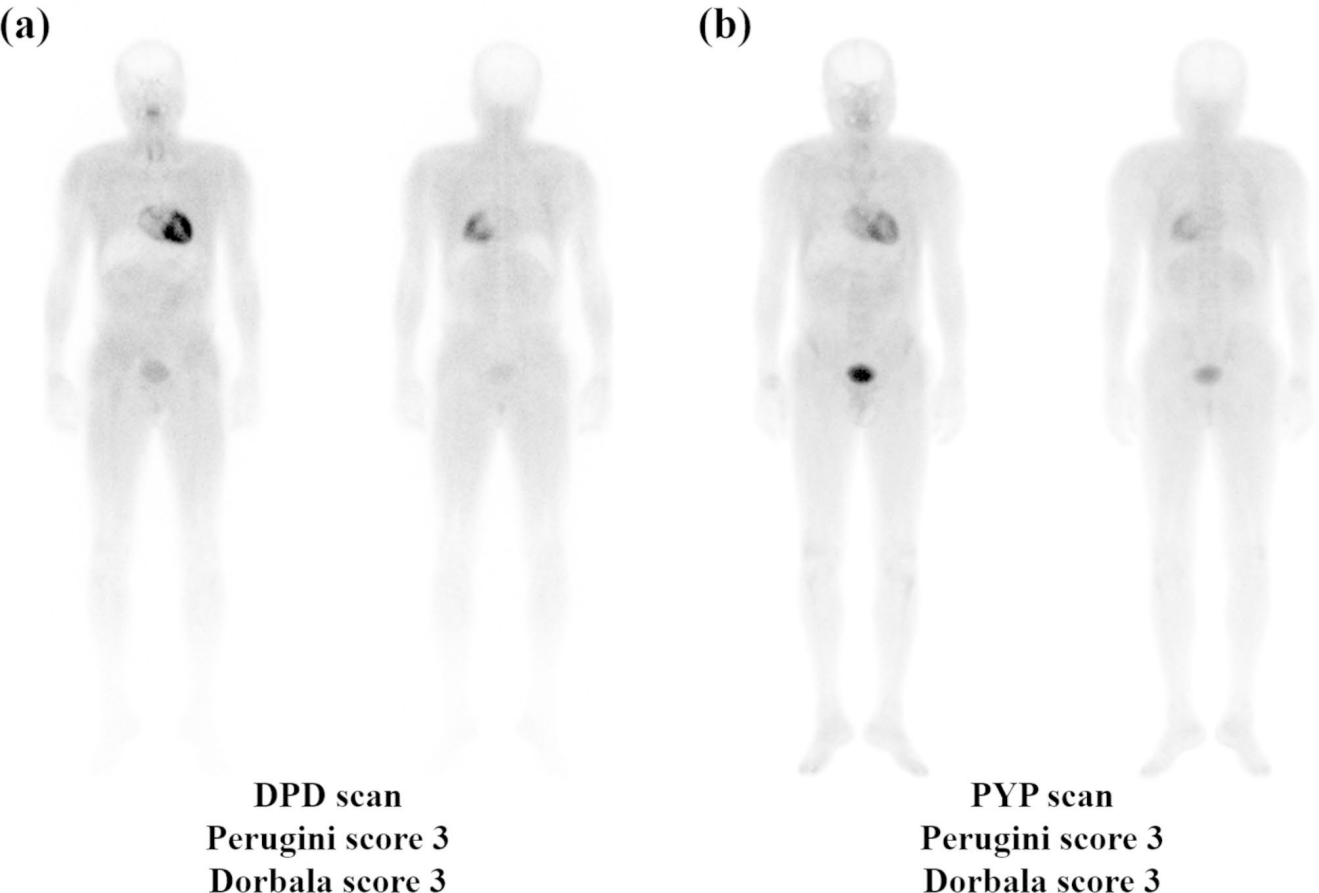
A representative case with the same visual score of 3 in **(a)** DPD and **(b)** PYP scans Abbreviations: DPD, technetium-99 m 3,3-diphosphono-1,2-propanodicarboxylic acid; PYP, technetium-99 m pyrophosphate.

**Fig. 3:**
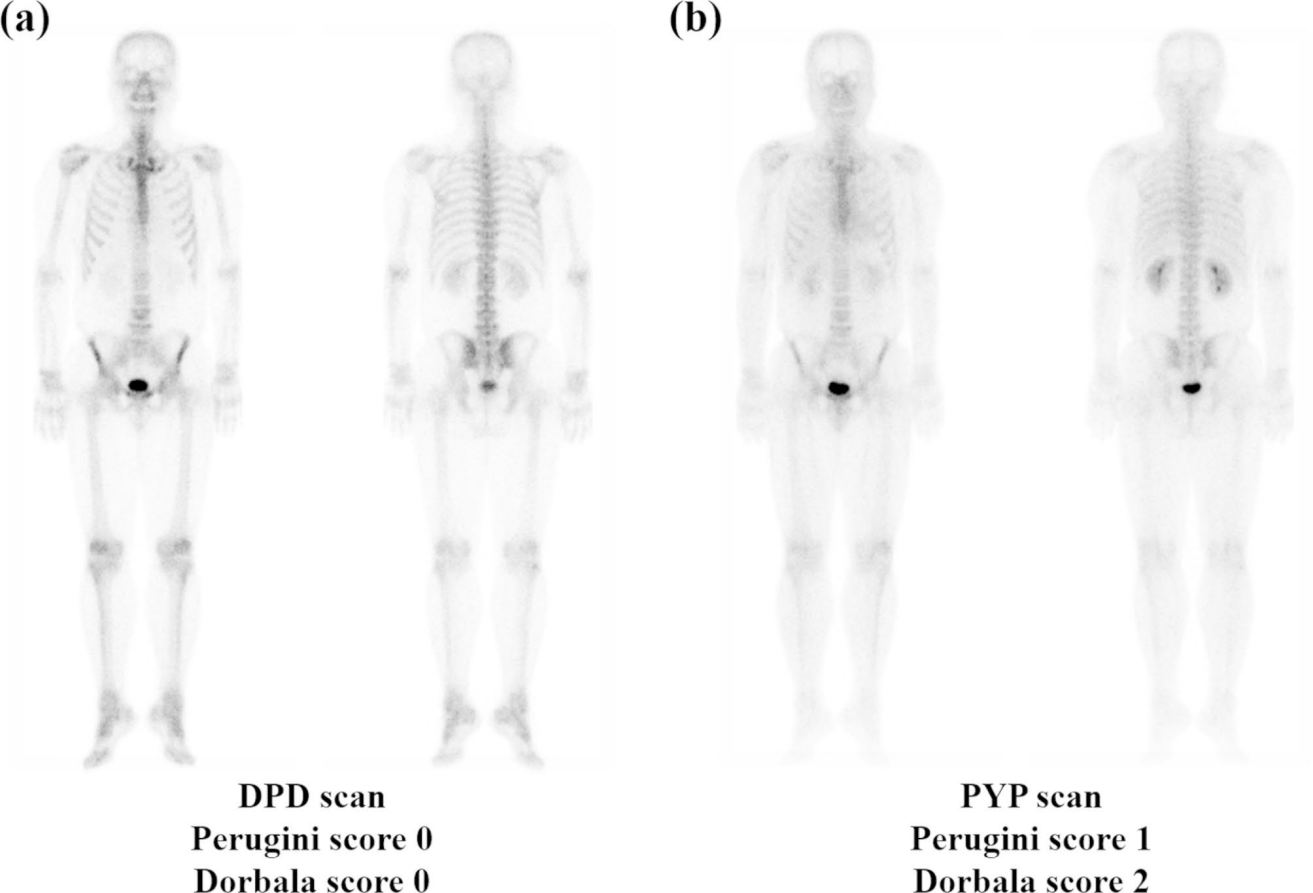
A representative case with different visual scores in the DPD and PYP scans. **(a)** In the DPD scan, both Perugini and Dorbala scores of the case patient were 0. **(b)** Perugini and Dorbala scores were higher in the PYP scan than the DPD scan, and the Dorbala score (Score 2) was higher than the Perugini score (Score 1) Abbreviations: DPD, technetium-99 m 3,3-diphosphono-1,2-propanodicarboxylic acid; PYP, technetium-99 m pyrophosphate

Significant correlations were observed in all retention ratios (H/WBp, H/P, H/S, and H/CL ratios) between DPD and PYP scans, and a significant correlation was observed in Perugini scores between DPD and PYP scans (Table 2). Among the four retention ratios, the strongest positive correlation was found in the H/CL ratio (P < 0.0001, rho = 0.952). Perugini scores on DPD and PYP scans showed a significant correlation (P = 0.0017, rho = 0.852), whereas Dorbala scores did not show a significant correlation (P = 0.2092, rho = 0.435). Six (60%) patients had no difference in Perugini and Dorbala scores between DPD and PYP scans (Fig. 2, Additional file 1: Table S1). In three patients, Perugini scores in the DPD scan differed from those in the PYP scan; two had Perugini scores that were one higher in the PYP scan than those in the DPD scan, and one had a Perugini score that decreased by one in the PYP scan compared to the DPD scan. Similarly, Dorbala scores of the DPD scans of three patients were different than Dorbala scores of the PYP scan; two had Dorbala scores that were two higher in the PYP scan than those in the DPD scan, and one had a Dorbala score that decreased by one in the PYP scan compared to the DPD scan. Dorbala system scores were relatively greater than those of Perugini system scores in PYP scans and DPD scans. In addition, compared to DPD scans, there were relatively more cases of higher visual scores in PYP scans than lower visual scores (Fig. 3). Moreover, there was one patient with a visual score of 0 in the Perugini and Dorbala systems based on the DPD scan, whereas there were no patients with a visual score of 0 in the Perugini and Dorbala systems in the PYP scan. In the Perugini system, eight patients (80%) had a visual score of 2 or higher in the DPD scan, and nine patients (90%) had a visual score of 2 or higher in the PYP scan. Similarly, in the Dorbala system, a visual score of 2 or higher on the DPD scan was found in eight patients (80%), and all patients had a visual score of 2 or higher on the PYP scan. There was no significant difference between the four retention ratios, two visual scores, WB tracer retention, or heart tracer retention between DPD and PYP scans.

**Table 2:** Scintigraphic findings in the DPD and PYP scans

		DPD scan	PYP scan			
		Median (range) or number of patients	Median (range) or number of patients	P value^†^	rho^‡^	P value^‡^
WB tracer retention		2,534,500 (1,730,000–2,905,000)	3,256,000 (1,590,000–4,466,000)	0.0696	0.612	0.0600
Heart tracer retention		117,892 (7,900 − 364,705)	163,222 (69,068–236,805)	0.3258	0.600	0.0667
Retention ratio	H/WBp	0.062 (0.006–0.157)	0.068 (0.034–0.092)	0.7052	0.841	0.0023^*^
	H/P	0.827 (0.095–2.649)	0.948 (0.520–1.552)	0.3845	0.879	0.0008^*^
	H/S	0.920 (0.054–2.727)	0.749 (0.349–1.236)	0.4057	0.915	0.0002^*^
	H/CL	20.646 (7.335–40.264)	21.133 (9.603–32.970)	0.7624	0.952	< 0.0001^*^
Visual score	Perugini score (0/1/2/3)	1/1/2/6	0/1/4/5	0.8996	0.852	0.0017^*^
	Dorbala score (0/1/2/3)	1/1/0/8	0/0/2/8	0.8287	0.435	0.2092

Abbreviations: DPD, technetium-99 m 3,3-diphosphono-1,2-propanodicarboxylic acid; PYP, technetium-99 m pyrophosphate; WB, whole-body; H/WBp, heart/whole-body profile; H/P, heart/pelvis; H/S, heart/skull; H/CL, heart/contralateral lung. ^*^ P < 0.05. ^†^ Mann-Whitney U test. ^‡^ Spearman’s correlation

### Correlations between visual scores and heart retention ratios in DPD and PYP scans

More frequent significant correlations between visual scores and heart retention ratios were observed in DPD scans than in PYP scans, and significant correlations between visual scores and heart retention ratios were observed more frequently in the Perugini system than in the Dorbala system (Table 3). DPD Perugini and DPD Dorbala scores were found to have significant correlations with all DPD retention ratios, and the DPD Perugini score had stronger correlations with DPD retention ratios than the DPD Dorbala score. PYP Perugini scores showed significant correlations with PYP H/WBp (P value = 0.0010, rho = 0.871), PYP H/P (P = 0.0082, rho = 0.777), and PYP H/S ratios (P = 0.0010, rho = 0.871), but no significant correlations with PYP H/CL ratio (P = 0.1102). PYP Dorbala scores had no significant correlations with any PYP retention ratios.

**Table 3:** Correlations between visual scores and heart retention ratios

Visual score	Retention ratio	rho^†^	P value^†^	Visual score	Retention ratio	rho^†^	P value^†^
DPD Perugini	DPD H/WBp	0.890	0.0006^*^	PYP Perugini	PYP H/WBp	0.871	0.0010^*^
	DPD H/P	0.829	0.0030^*^		PYP H/P	0.777	0.0082^*^
	DPD H/S	0.884	0.0007^*^		PYP H/S	0.871	0.0010^*^
	DPD H/CL	0.761	0.0106^*^		PYP H/CL	0.536	0.1102
DPD Dorbala	DPD H/WBp	0.705	0.0228^*^	PYP Dorbala	PYP H/WBp	0.435	0.2087
	DPD H/P	0.701	0.0240^*^		PYP H/P	0.348	0.3242
	DPD H/S	0.701	0.0240^*^		PYP H/S	0.348	0.3242
	DPD H/CL	0.683	0.0294^*^		PYP H/CL	0.261	0.4662

Abbreviations: DPD, technetium-99 m 3,3-diphosphono-1,2-propanodicarboxylic acid; PYP, technetium-99 m pyrophosphate; H/WBp, heart/whole-body profile; H/P, heart/pelvis; H/S, heart/skull; H/CL, heart/contralateral lung. ^*^ P < 0.05. ^†^ Spearman’s correlation

### Associations between scintigraphic parameters and clinical variables in DPD and PYP scans

DPD parameters showed more frequent significant associations with clinical variables than PYP parameters (Table 4). Significant associations were found between 20 DPD parameters and clinical variables, whereas only nine significant associations were found between PYP parameters and clinical variables. Significant differences were observed between eight scintigraphic parameters according to EMG abnormalities, and six of eight scintigraphic parameters (75%) were DPD parameters. Significant differences were observed in eight scintigraphic parameters according to diastolic dysfunction assessed by EchoCG, and six out of eight scintigraphic parameters (75%) were DPD parameters. Patients with diastolic dysfunction in EchoCG had significantly higher scintigraphic parameters. Furthermore, age and NT-proBNP level were positively correlated with six and five scintigraphic parameters, respectively. On the contrary, eGFR had significant negative correlations with DPD Dorbala score and PYP H/CL ratio. In addition, Dorbala parameters had more frequent significant associations with clinical variables than Perugini parameters, and five Dorbala and three Perugini scores had significant associations with clinical variables. DPD Dorbala scores had significant correlations with age, eGFR, and NT-proBNP, and significant differences were observed in DPD Dorbala scores according to EchoCG and EMG. The PYP Perugini score had a significant positive correlation with NT-proBNP, and significant differences were observed in DPD Perugini scores according to EchoCG and EMG.

**Table 4:** Significant associations between scintigraphic parameters and clinical variables in DPD and PYP scans

Scintigraphy	Scintigraphic parameter	Clinical variable	rho^†^	P value
DPD	DPD H/WBp	Age	0.745	0.0133^*†^
		NT-proBNP	0.840	0.0023^*†^
		EchoCG		0.0356^*‡^
		EMG		0.0356^*‡^
	DPD H/P	EchoCG		0.0367^*‡^
		EMG		0.0367^*‡^
	DPD H/S	NT-proBNP	0.802	0.0053^*†^
		EchoCG		0.0367^*‡^
		EMG		0.0367^*‡^
	DPD H/CL	Age	0.726	0.0175^*†^
		NT-proBNP	0.673	0.0330^*†^
		EchoCG		0.0367^*‡^
		EMG		0.0367^*‡^
	DPD Perugini	EchoCG		0.0182^*‡^
		EMG		0.0182^*‡^
	DPD Dorbala	Age	0.696	0.0253^*†^
		eGFR	-0.701	0.0240^*†^
		NT-proBNP	0.701	0.0240^*†^
		EchoCG		0.0029^*‡^
		EMG		0.0029^*‡^
PYP	PYP H/WBp	Age	0.659	0.0384^*†^
		EchoCG		0.0367^*‡^
		EMG		0.0367^*‡^
	PYP H/S	Age	0.634	0.0489^*†^
		EchoCG		0.0367^*‡^
		EMG		0.0367^*‡^
	PYP H/CL	Age	0.696	0.0253^*†^
		eGFR	-0.685	0.0289^*†^
	PYP Perugini	NT-proBNP	0.724	0.0180^*†^

Abbreviations: DPD, technetium-99 m 3,3-diphosphono-1,2-propanodicarboxylic acid; PYP, technetium-99 m pyrophosphate; H/WBp, heart/whole-body profile; NT-proBNP, N-terminal pro brain natriuretic peptide; EchoCG, echocardiography; EMG, electromyography; H/P, heart/pelvis; H/S, heart/skull; H/CL, heart/contralateral lung; eGFR, estimated glomerular filtration rate. ^*^ P < 0.05. ^†^ Spearman’s correlation. ^‡^ Mann-Whitney U test

## Discussion

To the best of our knowledge, this is the first study to directly compare DPD and PYP scans in patients with CA. Although DPD and PYP are the two most commonly used radiotracers for ATTR CA [6], no studies have directly compared them. In this study, significant correlations were observed in all semi-quantitative heart retention ratios between DPD and PYP scans. More frequent significant correlations were observed between visual scores and heart retention ratios in DPD scans than in PYP scans. DPD parameters had more frequent significant associations with clinical variables than PYP parameters. This suggests that DPD scans better reflect the disease severity of CA than PYP scans. Visual scores were more commonly higher rather than lower in PYP scans than DPD scans. Therefore, PYP scintigraphy may be a more sensitive imaging modality for identifying CA involvement than DPD scintigraphy.

In this study, DPD scans reflected the disease severity of CA better than PYP scans. Twenty DPD parameters and nine PYP parameters showed significant associations with age, eGFR, NT-proBNP, EchoCG, and EMG, suggesting that DPD parameters express the severity of CA better than PYP parameters. Previous studies reported that DPD and PYP parameters showed significant associations with age, eGFR, NT-proBNP, EchoCG, and EMG. Ternacle et al. demonstrated that the detection rate of CA using endomyocardial biopsy or autopsy increased noticeably as age increased [17]. Löfbacka et al. found that DPD-based amyloid burden had a robust correlation with echocardiographic strain parameters [8], and Dorbala et al. reported that maximum SUV, mean SUV, and cardiac amyloid activity in PYP SPECT/CT had moderate positive correlations with left ventricle mass index [12]. Therefore, as age increases, the incidence and prevalence of CA increases and the disease worsens, which is believed to increase DPD and PYP uptake. In patients with biopsy-proven renal amyloidosis, serum creatinine was shown to have a significant positive correlation with log_10_%amyloid, and a significant negative correlation was observed between eGFR and log_10_%amyloid [18]. This previous study arrived at similar conclusions to our study and found that eGFR had a significant negative correlation with DPD Dorbala and PYP H/CL. Coutinho et al. demonstrated that cardiac DPD uptake was correlated with NT-proBNP [19]. In addition, Peskó et al. reported that diastolic dysfunction was a common echocardiographic feature in CA [20], and Harapoz et al. found that quantitative DPD uptake had good correlations with transthoracic EchoCG parameters including left ventricle (LV) average wall thickness, LV indexed mass, and LV global longitudinal strain [21]. In a previous study, patients with a positive PYP SPECT had significantly higher interventricular septum thickness, significantly lower LVEF, and lower stroke volume index than those with a negative PYP SPECT [22]. As in previous studies, DPD and PYP parameters in this study showed significant positive correlations with NT-proBNP, and patients with diastolic dysfunction in EchoCG had significantly higher scintigraphic parameters than those without diastolic dysfunction. Furthermore, patients with sympathetic postganglionic sudomotor dysfunction in EMG were observed to have significantly higher DPD and PYP parameters than those without this sympathetic dysfunction. Pinto et al. suggested that myopathy can be an early manifestation of wide-type ATTR amyloidosis; diffuse skeletal muscle DPD uptake suggests that skeletal muscle may be a common site for amyloid deposition in patients with ATTR CM [23]. As in previous studies, DPD and PYP parameters were significantly associated with various clinical variables, and our results suggest that DPD scans reflect the disease severity of CA better than PYP scans.

Compared to DPD scintigraphy, PYP scintigraphy seems to be more sensitive at identifying CA involvement. In previous studies, both DPD and PYP scans were shown to have high sensitivity and specificity for myocardial uptake in patients with pathologically confirmed ATTR CM [24]. In the current study, one patient had Perugini and Dorbala visual scores of 0 in the DPD scan, whereas both Perugini and Dorbala scores were higher in the PYP scan. The proportion of patients with a visual score of 2 or higher, which indicates moderate to severe involvement of CA, was higher in PYP scans than DPD scans. More patients had higher visual scores rather than lower visual scores in PYP scans than DPD scans. Together, these findings suggest that PYP scans may be more sensitive for identifying CA involvement than DPD scans.

There were more frequent significant correlations between radiotracers and between visual scores and heart retention ratios using the Perugini system than the Dorbala system, whereas the Dorbala system reflected the disease severity of CA better than the Perugini system. Between DPD and PYP scans, Perugini scores showed significant correlations, but Dorbala scores showed no significant correlations. This is because the changes in visual scores of the Dorbala system were relatively large compared to those in the Perugini system. There were good correlations between Perugini visual scores and retention ratios in both DPD and PYP scans. On the contrary, there were good correlations between Dorbala visual scores and retention ratios only in DPD scans. In other words, significant correlations between visual scores and heart retention ratios were observed more frequently in the Perugini system than in the Dorbala system. These results provide evidence that there are better correlations between Perugini visual scores and heart retention ratios than Dorbala visual scores and heart retention ratios. However, contrary to the results of this study, a previous study of patients who underwent DPD scintigraphy and SPECT/CT with suspicion of CA reported that Dorbala visual scores were more significantly associated with H/CL and H/WB ratios than Perugini visual scores [13]. Therefore, these findings should be validated in further studies. In our study, Dorbala parameters showed more frequent significant associations with clinical variables than Perugini parameters, suggesting that the Dorbala system reflects the disease severity of CA better than the Perugini system. Our results support the use of the Dorbala system as the consensus standard diagnostic system for CA based on radionuclide imaging [15].

This study has several limitations. First, it was a retrospective single-center study, and the small sample size likely resulted in insufficient statistical power. Therefore, it is necessary to verify the results of this study in a prospective, large-scale multi-center study. Second, most of the enrolled patients in this study had ATTR CA (90%), whereas only a single AL CA patient (10%) was enrolled, limiting the generalizability of our findings. Third, our study used only DPD and PYP planar scintigraphy, and there was no direct comparison between DPD and PYP SPECT/CT. Asif et al. suggested that planar image-derived visual scores, while showing outstanding accuracy for SPECT myocardial uptake, were misclassified in a small proportion of patients (2.3%) [25]. Therefore, the DPD and PYP scintigraphy findings in this study need to be verified in future comparative studies of DPD and PYP SPECT/CT.

## Conclusions

DPD scintigraphy may reflect the disease severity of CA better than PYP scintigraphy, whereas PYP scintigraphy may be a more sensitive imaging modality for identifying CA involvement. In addition, Perugini visual scores and heart retention ratios showed stronger correlation in both DPD and PYP scans than Dorbala visual scores and heart retentions ratios, but the Dorbala system may reflect the disease severity of CA better than the Perugini system.

## Electronic supplementary material

Below is the link to the electronic supplementary material.

Supplementary Material 1

## Data Availability

The data presented in this study are available on request from the corresponding author.

## Abbreviations

CA: Cardiac amyloidosis
CM: Cardiomyopathy
ATTR: Amyloid transthyretin
AL: Amyloid immunoglobulin light-chain
ECG: Electrocardiogram
EchoCG: Echocardiography
CMR: Cardiovascular magnetic resonance
DPD: Technetium-99m 3,3-diphosphono-1,2-propanodicarboxylic acid
PYP: Technetium-99m sodium pyrophosphate
SPECT: Single-photon emission computed tomography
CT: Computed tomography
NT-proBNP: N-terminal pro brain natriuretic peptide
SUV: Standardized uptake value
H/WB: Heart/whole-body
ROI: Region of interest
H/WBp: Heart/whole-body profile
H/P: Heart/pelvis
H/S: Heart/skull
H/CL: Heart/contralateral lung
WB: Whole-body
eGFR: Estimated glomerular filtration rate
EMG: Electromyography
TTR: Transthyretin
LVEF: Left ventricular ejection fraction
LV: Left ventricle
