# Supplementary material for: Direct comparison of diagnostic and clinical values between Tc-99 m DPD and Tc-99 m PYP scintigraphy in patients with cardiac amyloidosis
Source: BMC Med Imaging. 2023 Jul 17;23:92. doi: 10.1186/s12880-023-01054-x (PMC10353145; doi:10.1186/s12880-023-01054-x)
Supplement: Supplementary file 1 — Supplementary Material 1 [file 12880_2023_1054_MOESM1_ESM.docx]

**Table S1** Perugini and Dorbala scores in DPD and PYP scans for each study patient.

|  | Perugini score | | Dorbala score | |
| --- | --- | --- | --- | --- |
|  | DPD | PYP | DPD | PYP |
| Patient 1 | 3 | 3 | 3 | 3 |
| Patient 2 | 1 | 2 | 1 | 3 |
| Patient 3 | 3 | 3 | 3 | 3 |
| Patient 4 | 2 | 2 | 3 | 3 |
| Patient 5 | 3 | 3 | 3 | 3 |
| Patient 6 | 3 | 3 | 3 | 3 |
| Patient 7 | 2 | 2 | 3 | 2 |
| Patient 8 | 0 | 1 | 0 | 2 |
| Patient 9 | 3 | 3 | 3 | 3 |
| Patient 10 | 3 | 2 | 3 | 3 |

Abbreviations: DPD, technetium-99m 3,3-diphosphono-1,2-propanodicarboxylic acid; PYP, technetium-99m pyrophosphate.
